# Causality between coffee intake and bone health: A Mendelian randomization study

**DOI:** 10.1097/MD.0000000000044533

**Published:** 2025-09-12

**Authors:** Heng Yang, Liling Yang, Zongping Li, Hongyuan Liu

**Affiliations:** a Department of Orthopaedics, Mianyang Central Hospital, School of Medicine, University of Electronic Science and Technology of China, Mianyang, Sichuan, PR China; b Department of Nephrology, Mianyang Central Hospital, School of Medicine, University of Electronic Science and Technology of China, Mianyang, Sichuan, PR China; c Department of Neurosurgery, Mianyang Central Hospital, School of Medicine, University of Electronic Science and Technology of China, Mianyang, Sichuan, PR China.

**Keywords:** coffee intake, Mendelian randomization, osteoarthritis, osteoporosis, rheumatoid arthritis

## Abstract

Observational studies have reported associations between coffee intake and osteoarthritis (OA), rheumatoid arthritis (RA), and osteoporosis (OP). However, it remains unknown whether these relationships are causal. In this study, we applied a 2-sample bidirectional Mendelian randomization (MR) analysis to comprehensively evaluate the causal association between coffee intake and OA, RA, and OP. Genetic data for OA (40,425 controls and 10,083 cases), RA (22,350 controls and 74,823 cases), OP (476,847 controls and 7751 cases), and coffee intake (263,464 Europeans) were obtained from the UK Biobank. The causal relationship between coffee intake and OA-, RA-, and OP-related traits was investigated using 2-sample MR analysis with a pooled dataset from genome-wide association studies. Inverse variance weighting (IVW) was used as the main outcome, whereas MR-Egger and weighted median were used to supplement the IVW estimates. Heterogeneity was assessed using Cochran *Q* statistic. Confounding-related single-nucleotide polymorphisms and single-nucleotide polymorphisms with *P* < .05 in MR-PRESSO analysis were excluded. There was no evidence of a causal relationship between coffee intake and the risk of RA (odds ratio [OR]: 0.84; 95% confidence interval [CI]: 0.24–2.93) using the IVW with random effect. Genetic predisposition to coffee intake was not associated with OA (OR: 1.21; 95% CI: 1.01–1.45) and OP (OR: 1; 95% CI: 0.99–1.0) using the main IVW method. Reverse MR analysis did not reveal evidence of a significant causal effect of OA, RA, and OP on coffee intake. Our findings do not support a causal relationship between coffee intake and the risk of OA, RA, and OP.

## 1. Introduction

Osteoarthritis (OA), rheumatoid arthritis (RA), and osteoporosis (OP) are common chronic bone diseases, and their incidence increases with aging. Although OA, RA, and OP are less likely to be directly forming a life in an individual’s life, the accompanying pain, depression, joint deformities, increased bone fragility, and bone deformities pose a serious burden on the patient’s finances and quality of life. Therefore, it is necessary to explore the factors associated with these chronic bone diseases.

Coffee contains many bioactive substances such as caffeine, caffeic acid, chlorogenic acid (CGA), heterocyclic amines, and acrylamide trigonelline, and it is the most widely consumed beverage in the world.^[1,2]^ Caffeine in coffee and the antioxidant properties of coffee have a positive effect on cardiovascular diseases, liver health, and cancer.^[3]^ Previous studies have shown that coffee inhibits tumor necrosis factor α, interleukin-1, and adenosine receptors, and increases osteoclast growth, thereby influencing the occurrence of OA.^[4]^ A meta-analysis from 2014 showed that coffee was a risk factor for RA.^[5]^ This may be related to the fact that caffeine in coffee promotes the production of interferon-γ in Th1 cells from RA patients in vitro through competitive inhibition of adenosine receptor A2a, which leads to T-cell activation and inflammation.^[6]^ In a prospective longitudinal cohort study of 121,701 women, Karlson showed that coffee intake was not associated with the risk of RA.^[7]^ A prospective cohort study of 57,053 individuals by Pedersen showed that coffee was not a risk factor for RA.^[8]^ Caffeic acid and CGA in coffee enhance the mechanical stress of the femoral diaphysis caused by the upregulation of tibia mineralization.^[9]^ CGA in the fruits of *Gardenia jasminoides* inhibits osteoclast function and leads to the inhibition of bone resorption.^[10,11]^ However, some studies have shown that coffee intake increases the risk of OP, especially in postmenopausal women, while caffeine in coffee reduces bone mineral density (BMD).^[12,13]^ However, the mechanism by which coffee increases the risk of OP remains unclear.

The studies on the relationship between coffee and the risk of OA, RA, and OP were mostly observational or experimental, and some of them have reported conflicting results. Thus, there is still uncertainty about the association between coffee and the risk of OA, RA, and OP.

Mendelian randomization (MR) is an innovative and popular form of analysis that integrates aggregated data from genome-wide association studies (GWAS) to assess the causal associations between inferred exposure and outcomes. Genetic variants are randomly arranged during fertilization and fixed after fertilization, with independence of confounding factors (such as environment, age, and sex). MR is similar to a randomized controlled trial, which can overcome the shortcomings of traditional epidemiological studies. However, MR has not yet been used to explore the association between coffee intake and bone health. Therefore, we conducted a 2-sample MR study to determine the causality between coffee intake and bone health.

## 2. Materials and methods

All analyses in this study were based on a publicly available GWAS aggregate. No additional ethical statement or informed consent was required.

### 2.1. Study design

**Figure 1.**
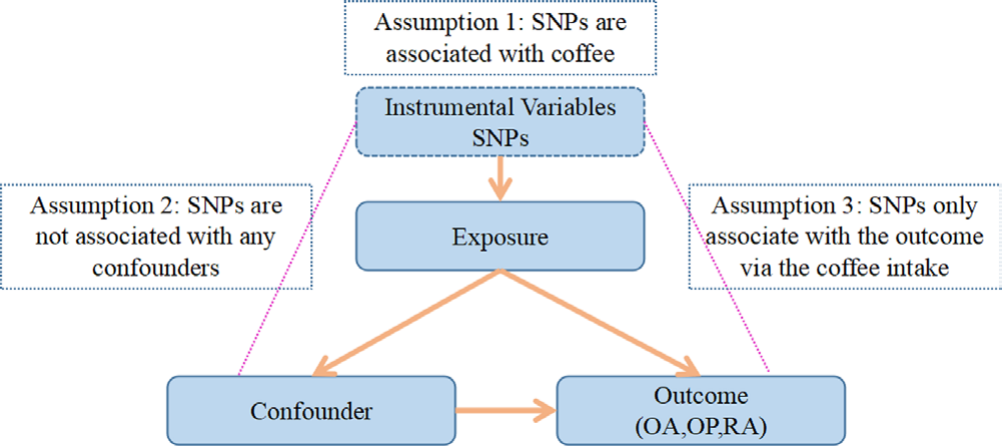
Conceptual framework for the Mendelian randomization analysis of coffee intake and OA, RA, and OP. OA = osteoarthritis, OP = osteoporosis, RA = rheumatoid arthritis.

A 2-sample MR analysis was performed to analyze the causal relationship between coffee intake and bone health. The research design process is shown in Figure 1. MR analyses need to meet the following 3 basic criteria: genetic variation is strongly related to exposure; genetic variation is not associated with potential confounders; and genetic variation should not affect results except through exposure modalities (Fig. 1).

### 2.2. Genetic instrument selection

Selected single-nucleotide polymorphisms (SNPs) significantly (*P* < 5 × 10^–8^) associated with exposure levels in the GWAS of the European population were selected as instrumental variables. To avoid collinearity among SNPs, related SNPs within each trait were excluded (linkage disequilibrium threshold, *r*^2^ < 0.001 and a cropping range of 10,000 kb). Potential confounders and outcomes were excluded from the PhenoScanner database, a platform for the relationship between genotypes and phenotypes. To avoid weak instrument bias, the *F*-statistic > 10 for each genetic instrument (SNP) was used as the threshold to ensure strong effect sizes in our MR analysis.

### 2.3. Data sources and data availability

The datasets generated and analyzed during the current study are available in publicly available GWAS aggregate. The GWAS summary statistics for coffee intake were based on a sample of 263,464 European pedigrees from the UK Biobank. The primary outcomes of this study were OA, RA, and OP. The OA data included 10,083 European ancestry cases and 40,425 European ancestry controls from a large multiethnic GWAS (GWAS ID: GCST005814). The GWAS data for the RA discovery cohort included 35,871 cases and 240,149 controls from Europe, Africa, East Asia, South Asia, etc. To ensure consistency with exposure data and reduce population stratification bias, the GWAS data from 22,350 cases and 74,823 controls with European ancestry samples were selected as the outcome data for this study. The GWAS data for OP were derived from genome-wide genotyping arrays of European ancestry, including 7751 cases and 476,847 controls.

### 2.4. MR analyses

Primary and main sensitivity analyses were performed using the random effects inverse variance weighting (IVW), which is the most commonly used method in MR analyses. MR-Egger (MRE) regression, weighted median (WM), and weighted mode were used to supplement the IVW estimates. “Leave-one-out” sensitivity analysis was used to further investigate the effects of outlying and/or pleiotropic genetic variation. Cochran *Q* statistic was used to test for heterogeneity among the genetic instruments. MRE intercept and MR-PRESSO were used as the secondary analysis to detect horizontal pleiotropy. If the estimates from these methods did not agree, the tightening tool *P*-value threshold was set and the MR analysis was performed again. *P* < .05 was considered statistically significant, and the Bonferroni method was used to adjust the *P*-value to correct for multiple comparisons. The MR analysis was performed using the R (version 4.2.1) (http://www.r-project.org) packages “TwoSampleMR” and “MR-PRESSO.”

## 3. Results

Nineteen SNPs as an instrument had an *F*-statistic higher than 10, thereby meeting the first hypothesis of the MR study (Table 1). The PhenoScanner data showed that 10 SNPs (rs574367, rs10188334, rs1877723, rs74904971, rs29647, rs16903275, rs2472297, rs56094641, rs476828, and rs56113850) were associated with RA and OA confounders and 9 SNPs (rs574367, rs10188334, rs1877723, rs74904971, rs29647, rs16903275, rs2472297, rs56094641, and rs476828) were associated with OP confounders. To satisfy the second hypothesis, these SNPs were eliminated when the corresponding analysis was performed. We used a series of sensitivity analyses to satisfy the third hypothesis.

**Table 1:** Characteristics of SNPs for habitual coffee intake.

SNP	CHR	Pos	EA	OA	Beta	SE	*P* value	N
rs574367	1	177,873,210	T	G	0.039524	0.0071104	2.72E‐08	263,464
rs1260326	2	27,730,940	T	C	−0.042454	0.005943	9.10E‐13	263,464
rs10188334	2	653,874	T	C	−0.04744	0.0078315	1.38E‐09	263,464
rs1877723	4	2,846,799	T	C	−0.036746	0.0063308	6.46E‐09	263,464
rs74904971	4	89,050,026	A	C	−0.064131	0.0091745	2.75E‐12	263,464
rs29647	5	170,560,546	C	T	−0.035603	0.0063284	1.85E‐08	263,464
rs34190000	5	7,381,260	T	G	−0.035148	0.0062971	2.38E‐08	263,464
rs16903275	5	87,950,543	A	C	0.050045	0.0080115	4.19E‐10	263,464
rs553108	6	31,840,455	A	G	0.033889	0.0059013	9.32E‐09	263,464
rs4410790	7	17,284,577	T	C	−0.12822	0.0060335	3.20E‐100	263,464
rs73075167	7	17,570,479	T	A	−0.054715	0.008997	1.19E‐09	263,464
rs34060476	7	73,037,956	G	A	0.070788	0.0085654	1.40E‐16	263,464
rs1057868	7	75,615,006	T	C	0.067087	0.0064143	1.33E‐25	263,464
rs73066852	12	11,276,541	G	A	0.046941	0.0080306	5.06E‐09	263,464
rs2472297	15	75,027,880	T	C	0.15404	0.0065528	3.42E‐122	263,464
rs56094641	16	53,806,453	G	A	0.039423	0.0059232	2.82E‐11	263,464
rs476828	18	57,852,587	C	T	0.052995	0.0068068	6.94E‐15	263,464
rs56113850	19	41,353,107	T	C	−0.042336	0.0058871	6.42E‐13	263,464
rs6062357	20	62,892,739	T	C	0.037018	0.0060214	7.86E‐10	263,464

CHR = chromosome, EA = effect allele, N = sample size, OA = other allele, *P* value = the value for the genetic association, Pos = position for SNP, SE = standard error, SNP = single-nucleotide polymorphism.

### 3.1. Causal relationship between coffee intake and the risk of OA

**Figure 2.**
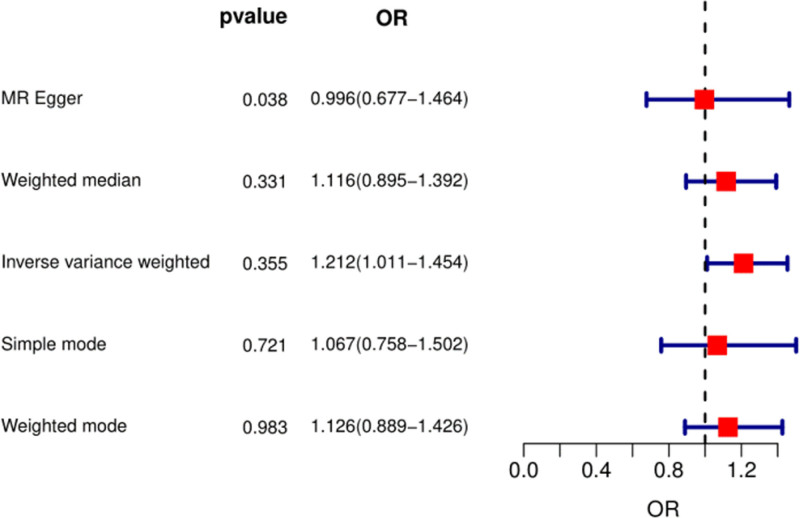
The causal effect of coffee intake on the risk of OA estimated using the MR study excluding 10 SNPs (rs574367, rs10188334, rs1877723, rs74904971, rs29647, rs16903275, rs2472297, rs56094641, rs476828, and rs56113850). MR = Mendelian randomization, OA = osteoarthritis, OR = odds ratio, SNP = single-nucleotide polymorphism.

The PhenoScanner database showed that 10 SNPs (rs574367, rs10188334, rs1877723, rs74904971, rs29647, rs16903275, rs2472297, rs56094641, rs476828, and rs56113850) were associated with OA confounders. After excluding these 10 SNPs, there was no causal relationship between genetically predicted coffee intake and OA. The odds ratio (OR) was 1.21 and 95% confidence interval (CI) was 1.01–1.45, using the IVW with MR random effects (Fig. 2). The WM approach (OR: 1.11; 95% CI: 0.89–1.39) did not support a causal relationship between coffee intake and the risk of OA (Fig. 2). The corresponding forest plot is shown in Figure 2. The *Q* statistic of the Cochran test showed that there was no heterogeneity: *Q* (df) = 5.7 (7) of MR-Egger method, *P* = .58. In addition, the MR-PRESSO analysis showed no signs of pleiotropy (*P* = .57).

### 3.2. Causal relationship between coffee intake and the risk of RA

**Figure 3.**
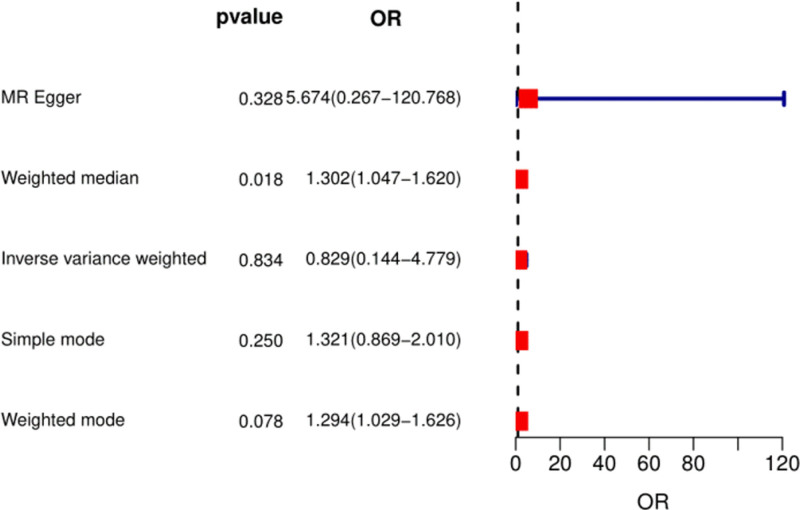
The causal effect of coffee intake on the risk of RA estimated using the MR study excluding 13 SNPs (rs574367, rs10188334, rs1877723, rs74904971, rs29647, rs16903275, rs2472297, rs56094641, rs476828, rs56113850, rs1260326, rs73075167, and rs34060476). MR = Mendelian randomization, OR = odds ratio, RA = rheumatoid arthritis, SNP = single-nucleotide polymorphism.

Ten SNPs associated with RA confounders were excluded from the PhenoScanner database (rs574367, rs10188334, rs1877723, rs74904971, rs29647, rs16903275, rs2472297, rs56094641, rs476828, and rs56113850). After stripping out the 10 SNPs, there was no evidence of a causal relationship between genetically predicted coffee intake and RA based on genetic instrumentation using the IVW with random effects (OR: 0.84; 95% CI: 0.24–2.93). The MR-PRESSO analysis showed signs of pleiotropy, excluded SNPs *P* < .05 in MR-PRESSO analysis (rs1260326, rs73075167, and rs34060476). After excluding the 3 SNPs, there was no evidence of a causal relationship between genetically predicted coffee intake and RA. The OR was 0.83 (95% CI: 0.14–4.78) using the IVW with MR random effects (Fig. 3). The WM approach (OR: 1.30; 95% CI: 1.05–1.62) did not support a causal relationship between coffee intake and the risk of RA (Fig. 3). The corresponding forest plot is shown in Figure 3. Although Cochran *Q* statistic indicated the presence of heterogeneity, it did not affect the results of the IVW and the conclusions were reliable.

### 3.3. Causal relationship between coffee intake and the risk of OP

**Figure 4.**
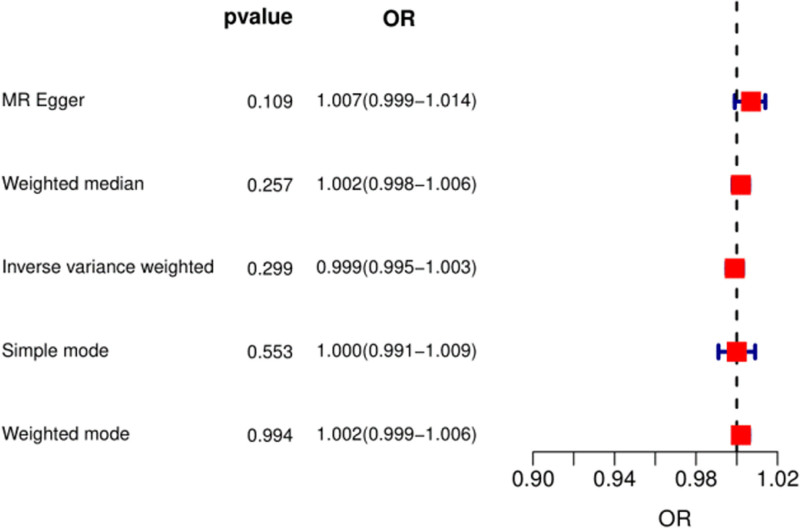
The causal effect of coffee intake on the risk of OP estimated using the MR study excluding 9 SNPs (rs574367, rs10188334, rs1877723, rs74904971, rs29647, rs16903275, rs2472297, rs56094641, and rs476828). MR = Mendelian randomization, OP = osteoporosis, OR = odds ratio, SNP = single-nucleotide polymorphism.

Nine SNPs (rs574367, rs10188334, rs1877723, rs74904971, rs29647, rs16903275, rs2472297, rs56094641, and rs476828) associated with OP confounders in the PhenoScanner database were excluded. The risk of OP did not increase with higher coffee consumption. In the IVW, the OR was 1 (95% CI: 0.99–1.0) (Fig. 4). The MRE and WM methods also confirmed that there was no causal relationship between coffee intake and OP, with *P* values of 0.3 and 0.26, respectively (Fig. 4). No heterogeneity was found in Cochran *Q* statistic (*Q* value (df) = 11.4 (8) of MR-Egger method, *P* = .18). The corresponding forest plot is shown in Figure 4. Leave-one-out analyses showed that none of the individual SNPs substantially affected the overall risk assessment. The MR-PRESSO analysis showed no evidence of pleiotropy (*P* = .06).

## 4. Discussion

MR can circumvent many of the limitations of traditional observational studies, and the large amount of GWAS data on multiple traits enhances the value of MR. We used this approach based on large GWAS data to analyze the causal relationship between coffee intake and the risk of OA, RA, and OP.

The association between coffee intake and the risk of OA, RA, and OP has been inconsistent in previous studies. OA is associated with the degradation of the collagen network, depletion of proteoglycans, and increased water content in articular cartilage. These structural changes reduce the biomechanical properties of cartilage and lead to dysfunction of the joints.^[14,15]^ Caffeine intake can negatively affect the physiology of joints and growth plate cartilage, thereby increasing the risk of OA.^[16]^ In a cross-sectional observational study in Korea, Bang et al showed that the prevalence of knee OA tended to increase with the increase in coffee consumption.^[17,18]^ However, some studies suggest that coffee consumption may be associated with a reduced risk of OA. CGA in coffee has the potential to inhibit the osteoclastogenesis, thereby reducing the risk of OA.^[19]^ Similarly, coffee’s antioxidant and anti-inflammatory properties may reduce the risk of OA.^[20]^ As described by Islam et al, some substances in coffee have antioxidant properties and reduce the risk of OA, while other components may increase the incidence of OA by antagonizing adenosine and promoting inflammation.^[21]^ Indeed, previous studies have shown that the role of coffee in OA is inconsistent and even divergent. Zhang et al conducted MR analyses demonstrating a significant genetic association between coffee intake and knee OA (OR: 2.03; 95% CI: 1.57–2.61; *P* < .001), but no significant association with hip OA (OR: 1.18; 95% CI: 0.53–2.65; *P* = .685).^[22]^ Furthermore, subgroup analyses stratified by coffee revealed no causal links with any part of OA.^[22]^ Notably, the study employed lenient instrument selection criteria. Zhang et al’s MR analysis revealed that genetically predicted 1% increase of coffee consumption was associated with an increased risk of overall OA (OR: 1.009, 95% CI: 1.003–1.016), knee OA (OR: 1.023, 95% CI: 1.009–1.038), self-reported OA (OR: 1.007, 95% CI: 1.003–1.011), but not hip OA (OR: 1.012, 95% CI: 0.999–1.024).^[23]^ However, in this study, the ORs were close to 1, raising concerns about their clinical significance despite statistical significance. Heterogeneity in coffee preparation methods and bioactive compound concentrations may introduce exposure misclassification. To address this limitation, we performed MR using GWAS summary statistics for total coffee intake. The primary IVW analysis showed no causal association between genetically predicted coffee intake and OA (OR: 1.21; 95% CI: 1.01–1.45; *P* = .36). The MR estimates reflect population-averaged effects with preserved causal directionality, providing valid etiological insights despite exposure heterogeneity.

RA is a chronic autoimmune disease, if left untreated, can cause disability and reduce quality of life. Although the etiology and pathogenesis of RA remain incompletely understood, dietary habits and environmental factors may play a significant role in disease development among genetically susceptible populations. As the world’s most widely consumed beverage, coffee has biological effects that could potentially interact with autoimmune mechanisms. However, current research findings regarding the association between coffee consumption and RA remain inconclusive. Coffee intake increases the risk of RA due to certain substances in coffee that are associated with the production of rheumatoid factor, inhibition of anti-inflammatory cytokines, inhibition of adenosine receptors, and oxidative stress.^[4,6,24]^ Substances in coffee and their derivatives can regulate the number of peripheral blood mononuclear cells to maintain immune function and play a protective role against RA.^[25,26]^ Bae et al reported the causal relationship between habitual coffee consumption (assessed through questionnaire-based frequency measures) and rheumatoid arthritis RA using MR analysis. Their findings did not support a causal association between coffee consumption and RA development.^[27]^ However, the questionnaire-based assessment may be subject to methodological limitations such as recall bias and non-differentiated categorization. In contrast, Lu et al reported a significant inverse correlation between sweetened coffee preference and RA (OR: 0.816; 95% CI: 0.667–0.997; *P* = .046).^[28]^ Notably, Lu et al’s study employed less stringent genome-wide significance thresholds (*P* < 8 × 10^–6^). To clarify this relationship, we conducted an MR analysis with stricter instrument selection criteria (*P* < 5 × 10^–8^) using expanded GWAS data. Our results demonstrated no causal relationship between coffee intake and RA (OR: 0.84; 95% CI: 0.14–4.78; *P* = .83).

OP, a prevalent chronic metabolic bone disorder, can lead to fragility fractures that significantly impair patients’ quality of life and mortality outcomes in advanced stages. While coffee consumption has been implicated in bone mineral density regulation, modifiable lifestyle factors may mitigate osteoporosis risk through targeted interventions. However, current evidence regarding the coffee and OP association remains contradictory. Caffeine can also adversely affect BMD by affecting adenosine receptors, thereby inhibiting bone formation and accelerating bone resorption.^[12]^ In studies of European populations, coffee was associated with a significantly lower BMD.^[29]^ In animal experiments, prenatal caffeine exposure induced delayed chondrogenesis in rats, and caffeine has been shown to promote osteoclast differentiation and decrease BMD in growing rats.^[13,30]^ However, according to animal studies, tannins in coffee improve the composition of articular cartilage in rats exposed to cadmium and improve the cartilage composition of the joints, suggesting that coffee may play a positive role in bone health.^[31,32]^ Wu et al conducted MR analysis and found no genetically causal association between coffee consumption and osteoporotic fracture risk.^[33]^ In contrast, Xu et al demonstrated a potential causal effect of decaffeinated coffee intake on OP (OR: 1.22; 95% CI: 1.05–1.42; *P* = .009).^[34]^ To avoid confounding risk factor violations and eliminate bias, here we used MR to analyze the causal relationship between coffee and the risk of OP. Confounding factors were excluded, and SNPs with *P* < .05 excluded in MR-PRESSO analysis were excluded. After adjusting the *P*-value using the Bonferroni method, we found that the IVW, WM, and MR-Egger results did not support a causal relationship between coffee intake and OP.

## 5. Strengths and limitations

The advantages of the 2-sample MR study include a larger sample size, summarizing genetic data, overcoming the limitations of traditional epidemiology (such as residual confounding and reverse causality bias), and being less time-consuming and costly than randomized controlled trials. However, this MR study has some unavoidable limitations. First, to reduce the effect of stratified populations, bias was limited to European populations, and our results do not apply to other populations. Second, valid information on coffee intake, such as coffee brewing method, type of coffee intake, and amount of coffee intake, was not assessed. These factors need to be taken into account in further analysis. Finally, we used summary statistics and could not accurately calculate sample overlap between exposures and outcomes.

## 6. Conclusion

Our analysis does not support a causal relationship between coffee intake and the risk of OA, RA, and OP.

## Acknowledgments

The authors want to thank all the consortia that provided the GWAS dataset available to the public.

## Author contributions

**Conceptualization:** Hongyuan Liu, Heng Yang.

**Data curation:** Hongyuan Liu, Liling Yang.

**Investigation:** Heng Yang.

**Methodology:** Hongyuan Liu, Heng Yang.

**Resources:** Hongyuan Liu, Liling Yang.

**Software:** Hongyuan Liu, Heng Yang, Liling Yang, Zongping Li.

**Supervision:** Hongyuan Liu, Zongping Li.

**Writing – original draft:** Heng Yang.

**Writing – review & editing:** Hongyuan Liu.
